# Comparative Genome Analysis of *Mycobacterium avium* Revealed Genetic Diversity in Strains that Cause Pulmonary and Disseminated Disease

**DOI:** 10.1371/journal.pone.0071831

**Published:** 2013-08-21

**Authors:** Kei-ichi Uchiya, Hiroyasu Takahashi, Tetsuya Yagi, Makoto Moriyama, Takayuki Inagaki, Kazuya Ichikawa, Taku Nakagawa, Toshiaki Nikai, Kenji Ogawa

**Affiliations:** 1 Department of Microbiology, Faculty of Pharmacy, Meijo University, Nagoya, Japan; 2 Department of Pharmacy, Kainan Hospital Aichi Prefectural Welfare Federation of Agricultural Cooperatives, Yatomi, Japan; 3 Department of Infectious Diseases, Center of National University Hospital for Infection Control, Nagoya University Hospital, Nagoya, Japan; 4 Department of Pharmacy, National Hospital Organization, Nagoya Medical Center, Nagoya, Japan; 5 Department of Pharmacy, Takayama Red Cross Hospital, Takayama, Japan; 6 Department of Pharmacy, Nagoya University Hospital, Nagoya, Japan; 7 Department of Clinical Research, National Hospital Organization, Higashinagoya National Hospital, Nagoya, Japan; 8 Department of Pulmonary Medicine, National Hospital Organization, Higashinagoya National Hospital, Nagoya, Japan; Institut de Pharmacologie et de Biologie Structurale, France

## Abstract

*Mycobacterium avium* complex (MAC) infection causes disseminated disease in immunocompromised hosts, such as human immunodeficiency virus (HIV)-positive patients, and pulmonary disease in persons without systemic immunosuppression, which has been increasing in many countries. In Japan, the incidence of pulmonary MAC disease caused by *M. avium* is about 7 times higher than that caused by *M. intracellulare*. To explore the bacterial factors that affect the pathological state of MAC disease caused by *M. avium*, we determined the complete genome sequence of the previously unreported *M. avium* subsp. *hominissuis* strain TH135 isolated from a HIV-negative patient with pulmonary MAC disease and compared it with the known genomic sequence of *M. avium* strain 104 derived from an acquired immunodeficiency syndrome patient with MAC disease. The genome of strain TH135 consists of a 4,951,217-bp circular chromosome with 4,636 coding sequences. Comparative analysis revealed that 4,012 genes are shared between the two strains, and strains TH135 and 104 have 624 and 1,108 unique genes, respectively. Many strain-specific regions including virulence-associated genes were found in genomes of both strains, and except for some regions, the G+C content in the specific regions was low compared with the mean G+C content of the corresponding chromosome. Screening of clinical isolates for genes located in the strain-specific regions revealed that the detection rates of strain TH135-specific genes were relatively high in specimens isolated from pulmonary MAC disease patients, while, those of strain 104-specific genes were relatively high in those from HIV-positive patients. Collectively, *M. avium* strains that cause pulmonary and disseminated disease possess genetically distinct features, and it suggests that the acquisition of specific genes during strain evolution has played an important role in the pathological manifestations of MAC disease.

## Introduction

Many species of nontuberculous mycobacteria (NTM) are found in a variety of habitats, including natural water, water distribution systems, bathrooms, soil, and household dust [1]–[4]. Unlike tuberculosis, direct transmission via human-to-human contact is rare in NTM infection. Instead, NTM infection is thought to occur via exposure to aerosols containing mycobacteria in the environment, although the exact source has not been specified. In Japan, approximately 90% of NTM infections are caused by *Mycobacterium avium* complex (MAC, 70–80%) and *M. kansasii* (10–20%) [5].

MAC infection can be attributed to two closely related organisms, *M. avium* and *M. intracellulare*. *M. avium* comprises four subspecies that infect specific hosts: *M. avium* subsp. *avium* and *M. avium* subsp. *silvaticum* are avian pathogens; *M. avium* subsp. *hominissuis* is found in the environment, humans, and pigs; and *M. avium* subsp. *paratuberculosis* causes disease in livestock and wildlife. It is generally considered that *M. avium* subsp. *hominissuis* is responsible for MAC disease [6]. The presence (or absence) of specific insertion sequences (*IS1245*, *IS900,* and *IS901*) can be used to distinguish subspecies [7], but today, sequencing of the *hsp65* gene, one of the house keeping genes of *M. avium,* provides more accurate information for subspecies identification [8].

*M. avium* is an opportunistic pathogen that causes generalized disseminated disease in immunocompromised patients, such as human immunodeficiency virus (HIV)-positive patients. *M. avium* infection spread with acquired immunodeficiency syndrome (AIDS) in the 1980’s. In contrast to HIV-associated disseminated MAC disease, pulmonary MAC disease in immunocompetent persons is caused by *M. intracellulare* and *M. avium*, and their prevalence varies by country. In Japan, the incidence of pulmonary MAC disease caused by *M. avium* is about 7 times higher than that caused by *M. intracellulare* [5]. In recent years, pulmonary MAC disease producing lesions in the lingular segments and middle lobe is increasing in middle-aged to elderly females with no underlying disease in many countries [9]. The prevalence of NTM lung disease in Japan increased dramatically from 0.82 per 100,000 population in 1971 to 5.9 per 100,000 population in 2001 [10], and the current rate is estimated as 8.0–10.0 per 100,000 population. This rate is substantially higher than the rates seen in the United State and Europe [11]–[13].

It is likely that bacterial factors, as well as host-related risk factors, are associated with the establishment of pulmonary MAC disease. Although results are inconclusive, several studies have investigated the following possible host-related risk factors: decreases in the levels of estrogen [14], a major female sex hormone, and the presence of polymorphisms in *NRAMP1* (encoding natural resistance-associated macrophage protein 1) [15] and *MICA* (encoding major histocompatibility complex class I chain–related A) [16]. On the other hand, little is known about bacterial factors.

The mechanisms of the pathogenicity of mycobacteria involve the following: prevention of maturation of pathogen-containing phagosomes in host macrophages [17]; production of enzymes, such as catalase [18], that remove reactive oxygen species; and synthesis of mycobactin that serves as a siderophore to effectively acquire iron necessary for bacterial growth [19]. In addition to these mechanisms, mycobacteria are also equipped with mechanisms for invading host cells [20].

In this study, we carried out whole-genome sequencing on the previously unreported *M. avium* subsp. *hominissuis* strain TH135 isolated from a HIV-negative patient with pulmonary MAC disease, and we performed comparative analysis between genomes of strain TH135 and strain 104 isolated from an AIDS patient with MAC disease to examine the bacterial factors that affect the establishment of pulmonary disease caused by *M. avium*.

## Materials and Methods

### Bacterial Strains, Growth Condition, and Genomic DNA Isolation

The clinical isolates used in this study comprised 35 *M. avium* strains including the genome analysis strain TH135 recovered from the sputa of HIV-negative patients with pulmonary MAC disease at the National Hospital Organization, Higashinagoya National Hospital in Japan from 2004 to 2008. In addition, 28 *M. avium* clinical isolates derived from blood of HIV-positive patients with disseminated MAC disease were provided by the National Center for Global Health and Medicine, formerly called the International Medical Center of Japan. The subspecies of *M. avium* clinical isolates was identified as *M. avium* subsp. *hominissuis* by sequence analysis of the 3′ fragment of the *hsp65* gene [8]. The organism was grown in Middlebrook 7H9 liquid medium supplemented with 10% oleic acid/albumin/dextrose/catalase enrichment (Difco Laboratories, Detroit, MI) at 37°C. Genomic DNA was extracted with a Qiagen kit (Qiagen Inc., Valencia, CA) according to the manufacturer’s instructions.

### Genome Sequencing and Annotation

The genome sequence of *M. avium* subsp. *hominissuis* strain TH135 was determined by combining the technology of two genome sequencers: 454 GS FLX (Roche, Mannheim, Germany); and Hiseq 2000 (Illumina, CA). The genomic DNA was first sequenced using the Hiseq with 101-bp paired-end library (80,119,704 reads, 1,600-fold genome coverage), and sequence reads were assembled using Velvet (version 1.2.07). Gaps between the contigs were closed by mapping with FLX 8-kb paired-end reads (295,431 reads, 14-fold genome coverage) obtained using GS De Novo Assembler (version 2.7). The sequence obtained by the Hiseq was further compared with data obtained by the FLX, and unmatched sequences and gap sequences in the scaffolds were filled by PCR amplification followed by Sanger sequencing. The genome sequence was automatically annotated using the Microbial Genome Annotation Pipeline [21] and corrected manually using *in silico* Molecular Cloning Genomics Edition (IMCGE) software [22].

### Bioinformatics Analysis

Protein function was assigned based on a BLASTP similarity search against the NCBI ‘nr’ (non-redundant protein) database. Transfer RNA (tRNA) and ribosomal RNA (rRNA) were predicted using a tRNAscan-SE 1.23 [23] and RNAmmer 1.2 [24], respectively. Insertion sequence (IS) elements were identified using the IS-Finder [25], and the microbial genome database (MBGD) was used to detect conserved gene clusters [26].

### Comparative Genomic Analysis

Comparative genomic analysis was performed with *M. avium* subsp. *hominissuis* strain 104 (GenBank accession no. NC_008595), which was derived from an AIDS patient with MAC disease [27]. IMCGE software was used for data management and for visualization of genomic features. Multiple whole-genome alignments were performed using Mauve software, which was designed for the identification and alignment of conserved genomic DNA in the presence of rearrangements [28]. Orthologues in *M. avium* strains TH135 and 104 were defined by bidirectional best-hit analysis between the genomes of both strains with a threshold of >90% amino acid identity and >60% aligned length coverage of a query sequence. The remaining coding sequences (CDS) without the characteristics of orthologues were defined as *M. avium* strain-specific CDSs.

### PCR Analysis and Sequence Analysis

Clinical isolates were cultured in 5 mL 7H9 liquid medium supplemented with 10% oleic acid/albumin/dextrose/catalase enrichment at 37°C for 1–2 weeks and then transferred to 25 mL of the same medium for further culture. The culture was centrifuged, and DNA was extracted using an InstaGene Matrix (Bio-Rad Laboratories, Hercules, CA) according to the manufacturer’s instructions. The PCR mixture (50 µL) was composed of DNA solution (5–50 ng), 1 U of AmpliTaqGold DNA polymerase (Applied Biosystems, Foster City, CA), 5 µL of 2 mM deoxynucleoside triphosphate mixture, 5 µL of 10×PCR buffer, 1 µL of each primer set at 25 µM, and dimethyl sulfoxide (Sigma-Aldrich) to a final concentration of 4%. The PCR primers used in this study are shown in Table S1. The PCR conditions were as follows: 1 cycle at 95°C for 10 min; 35 cycles at 94°C for 1 min, 55°C for 1 min, and 72°C for 1 min; and 1 cycle at 72°C for 7 min. The PCR products were electrophoresed with the TrackIt 50 bp DNA ladder (Invitrogen, San Diego, CA) in a 2% agarose gel (E-gel; Invitrogen). The resulting PCR products were purified using a GenElute PCR DNA purification kit (Sigma-Aldrich), and direct sequencing analysis was performed using the same primers as those used for PCR. The resulting nucleotide sequences were compared with the genomic sequence data for *M. avium* strain TH135 and strain 104. Accordingly, the presence of strain TH135- and 104-specific genes in clinical isolates was determined by the use of specific primers in PCR. The suitability of the present DNA samples for screening of clinical isolates with PCR was determined by amplification of the *hsp65* gene – the gene used to identify subspecies of *M. avium* in clinical isolates.

### Statistical Analysis

Data for the detection rate of specific genes in *M. avium* clinical isolates were analyzed statistically using Fisher’s exact test. *P* values of <0.05 were considered significant.

### Nucleotide Sequence Accession Number

The complete chromosome sequence of *M. avium* subsp. *hominissuis* strain TH135 has been deposited in DDBJ/EMBL/GenBank under accession no. AP012555.

## Results and Discussion

### General Genomic Features

**Figure 1:**
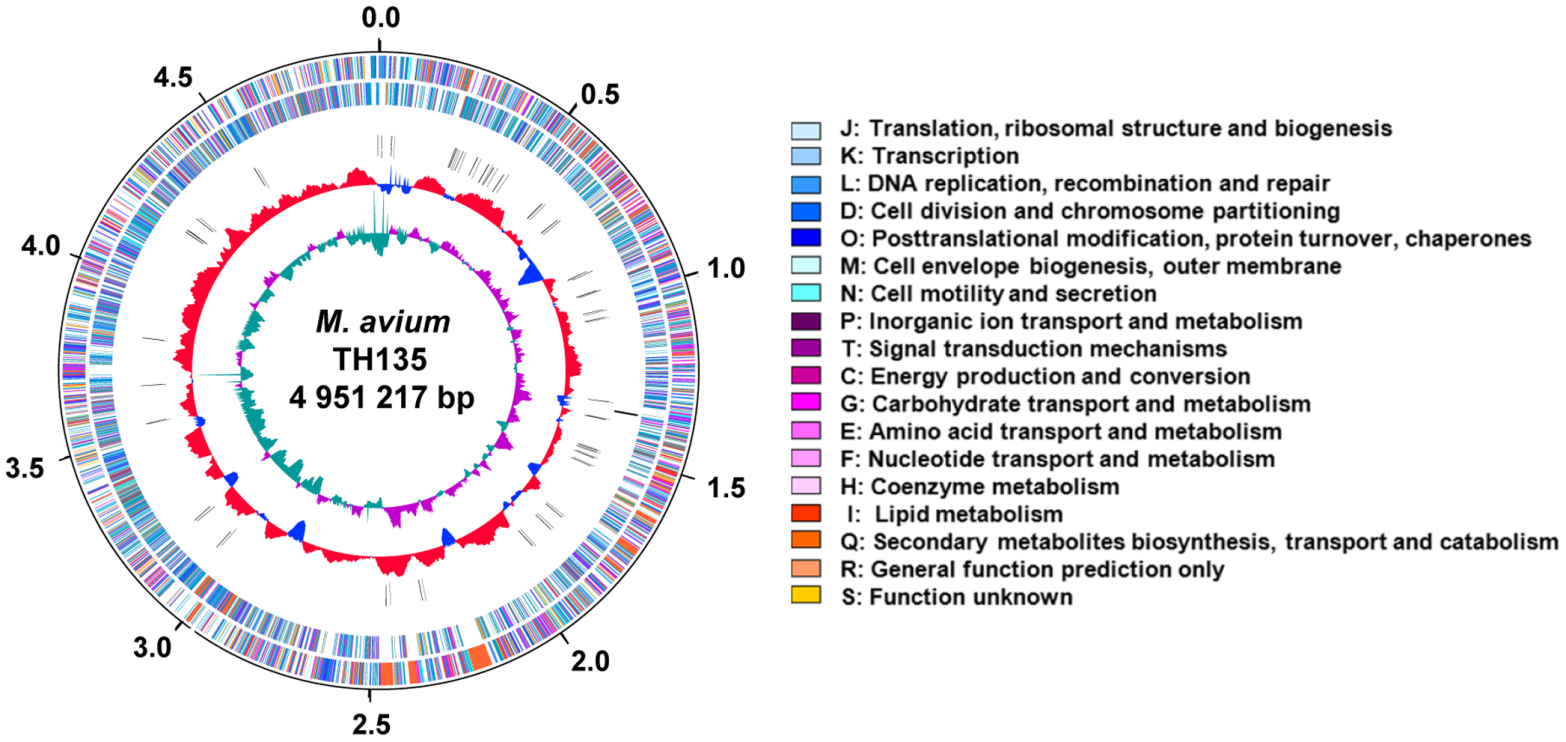
Circular representation of the *M. avium* strain TH135 genome. The scale is shown in base pairs (Mb), with zero representing the location of the *dnaA* gene. From the outside to the inside, the outer two circles show forward- and reverse-strand coding sequences (CDS), respectively. The third and fourth circles show rRNA operons and tRNA genes, respectively. The fifth circle shows the percentage of G+C in relation to the mean G+C of the chromosome. The sixth circle shows the GC skew (G – C)/(G+C). The color of each CDS was assigned according to the cluster of orthologous groups (COG) functional classification system [31]. The color of each COG family is shown in the figure.

To explore the bacterial factors that affect the establishment of pulmonary disease caused by *M. avium* subsp. *hominissuis*, we determined the whole genome sequence of the previously unreported *M. avium* strain TH135 isolated from a HIV-negative patient with pulmonary disease and compared it with the complete genome of *M. avium* strain 104 derived from an AIDS patient with MAC disease [27]. The general features of strain TH135 compared with those of strain 104 are presented in Table 1. The replication origin of the strain TH135 chromosome was deduced on the basis of the transition point in GC skew analysis and the presence of the *dnaA* gene accompanied by several DnaA boxes (Fig. 1). The genome was composed of a single circular chromosome of 4,951,217 bp with an average G+C content of 69.32%, 4,636 predicted CDS, 46 tRNA genes, and a single rRNA operon with the typical order of 16S, 23S, and 5S rRNA genes. The chromosome size of strain TH135 is 524,274 bp shorter than that of strain 104 (5,475,491 bp). Although both strains belong to the same subspecies, IS content is very different between the strains, and the strain 104 genome carries more IS elements than the strain TH135 genome. On the other hand, it is noteworthy that strain TH135 harbors five IS*Mav6* genes (MAH_0649, MAH_1321, MAH_2272, MAH_2945, and MAH_3485) that have 60 point mutations compared with a subspecies differentiation marker IS*901*, which is on the genomes of different subspecies*––M. avium* subsp. *avium* and *M. avium* subsp. *silvaticum* [29]. IS elements are thought to be one of the major players in prokaryote genome plasticity [30]. A greater number of IS elements indicates that the genome has undergone further structural variation during strain evolution.

**Table 1:** General features of the *M. avium* strain TH135 genome and comparison with *M. avium* strain 104.

Property	Strain TH135	Strain 104
Genome size, bp	4,951,217	5,475,491
G+C content, %	69.3	69.0
Protein coding, %	92.5	88.6
ORFs	4,636	5,120
Average gene length, bp	984	948
tRNAs	46	46
rRNA operons (16S-23S-5S)	1	1
Prophage elements, no.	0	1
IS, total no. of copies	30	129

### Comparative Study

**Figure 2:**
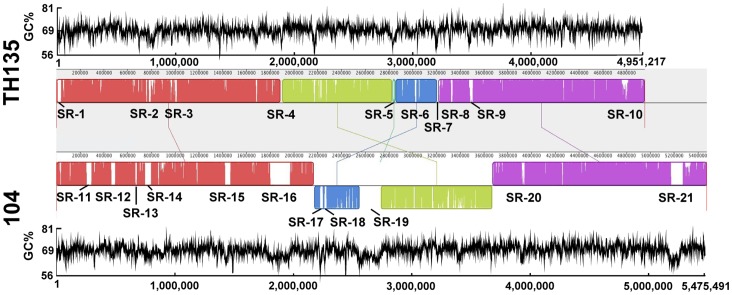
Whole genome alignment and G+C content of *M. avium* strain TH135 (upper) and 104 (lower). Representation of five local collinear blocks (LCBs) between chromosomal sequences of each strain generated by Mauve alignment software. The connecting lines between blocks indicate the location of each block in each genome. Each colored block represents a homologous region. LCBs drawn horizontally represent homology in the reverse strand of the chromosome. Gaps or white spaces within LCBs indicate the presence of strain-specific sequences. Specific region (SR)-1 to SR-10 in strain TH135 and SR-11 to SR-21 in strain 104 show strain-specific regions of over 10,000 bp in length.

Whole-genome alignment of both strains was carried out using Mauve software (Fig. 2). Although high conservation in both the sequence and gene order of strain TH135 and 104 was observed, there were gene insertions and two large inversions (green and blue blocks in Fig. 2). Gaps or white spaces within blocks indicate the presence of strain-specific sequences. On specific regions of over 10,000 bp in length, strain TH135 has 10 loci (specific region (SR)-1 to SR-10) and strain 104 has 11 loci (SR-11 to SR-21). Compared with strain TH135, the strain 104 genome possesses many specific regions of large size. Of these specific regions, many CDSs in SR-3, SR-10, and SR-19 were highly homologous to the CDSs in *M. paratuberculosis*, *M. parascrofulaceum,* and *M. intracellulare*, respectively (Table S2). Interestingly, many of these regions have low G+C content compared with the mean G+C content of the corresponding chromosome, which is an added sign of foreign origin (Fig. 2; Table S2 and Table S3). Furthermore, such specific regions are flanked by genes which encode integrases of phage origin and/or transposases derived from transposons (Table S2 and Table S3). In particular, region SR-14 of strain 104 was identified as a prophage insertion region flanked by phage insertion sites *attL* and *attR*. Also interestingly, a comparison of the genome of *M. avium* subsp. *paratuberculosis* (GenBank accession no. NC_002944), which causes disease in livestock and wildlife, and the genome of the present strains (TH135 and 104) revealed that regions SR-1–SR-10, but not SR-3, of strain TH135 and regions SR-11–SR-21 of strain 104 are present as specific regions in the strain TH135 and strain 104 genomes (data not shown). Taken together, these regions are likely to be inserted into chromosomes via horizontal gene transfer during strain evolution. Although strain TH135 and strain 104 show high genomic DNA sequence similarity (94.4% for strain TH135 and 86.6% for strain 104), both harbor many strain-specific regions, suggesting that both strains evolved independently from a common ancestor.

**Figure 3:**
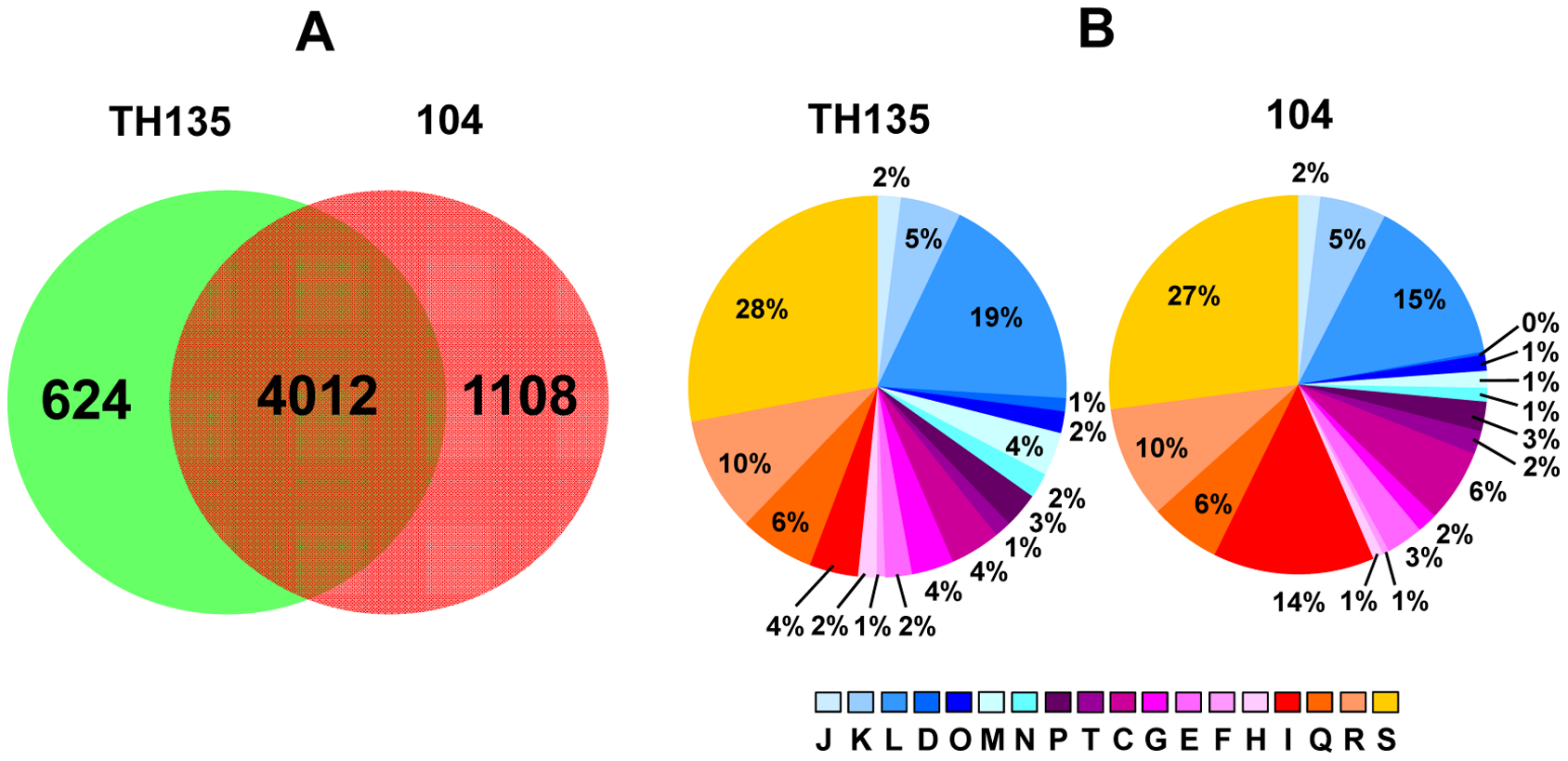
Venn diagram of *M. avium* strains TH135 and 104 genomes and comparison of COG functional categories between strain TH135- and 104-specific genes. (A) Venn diagram showing the distribution of shared orthologues and strain-specific genes between *M. avium* strains TH135 and 104. The Venn diagram was created with IMCGE software. Comparative analysis revealed that 4,012 genes are shared between the two strains, and 624 genes and 1,108 genes are unique to strains TH135 and 104, respectively. (B) Classification of specific genes of each strain based on COG functional categories. Each colored segment indicates the relative contribution of a functional category as a percentage of total COGs. The color of each COG family is shown in Figure 1.

The Venn diagram in Fig. 3A shows the distribution of shared orthologues and strain-specific genes between strains TH135 and 104. As shown in Fig. 3A, 4,012 genes were shared between the two strains, whereas the number of strain TH135- and 104-specific genes was 624 (13.5%) and 1,108 (21.6%), respectively. Furthermore, strain-specific ORFs are classified according to cluster of orthologous groups (COG) category (Fig. 3B) [31]. The relative contribution of COGs are generally similar between strain TH135-specific genes and strain 104-specific genes, and category S (function unknown) and category L (recombination and repair) genes were dominant in both strains. However, the relative contribution of category I (lipid metabolism) was 3.5-fold higher in strain 104-specific genes (14%) than in strain TH135-specific genes (4%), while that of category M (cell envelope and outer membrane biogenesis) was 4-fold higher in strain TH135-specific genes (4%) than in strain 104-specific genes (1%).

### Comparison of Virulence-associated Factors

We considered that differences in virulence-associated factors between strain TH135 and strain 104 might explain the different pathological manifestations of MAC disease caused by *M. avium*. Thus, we searched for such factors and compared them between the strains.

### Mammalian Cell Entry

Four mammalian cell entry (*mce*) operons (*mce1* to *mce4*), found in the genome sequence of *M. tuberculosis* H37Rv, are associated with the virulence of *M. tuberculosis* [20]. Each *mce* locus comprises two *yrbE* and six *mce* genes (*mceA* to *mceF*), which are homologous to the permeases and substrate-binding proteins of ABC transporters, respectively [32]. Although the precise mechanisms involving Mce proteins remain unclear, it was demonstrated that secreted Mce1A protein moves to the bacterial surface, thereby facilitating entry of mycobacteria into macrophages and HeLa cells, followed by subsequent survival [20], [33]. Like Mce1A protein, Mce3A and Mce3E are also involved in cellular uptake of mycobacteria [34], while Mce2A appears to have a distinct role [33]. The *mce4* operon is implicated in the uptake of cholesterol, which is an essential energy source for mycobacteria for its long-term survival in host cells [35]. Klepp et al. showed the possible involvement of *mce* operons of *M. smegmatis* in the maintenance of cell surface properties [36]. Furthermore, bioinformatics evidence suggests that some Mce proteins of the bacterial membrane contribute to the formation of beta barrel proteins serving as channels [37]–[39].

We found that the number of *mce* operons varies between strain TH135 (n = 7) and strain 104 (n = 9). The genomes of strain TH135 and 104 contained CDSs with a 52.0–84.3% sequence homology to Mce proteins encoded by operons *mce1*–*4* in the *M. tuberculosis* H37Rv genome (GenBank accession no. NC_000962) (Table S4). Comparison of *mce* family genes in strain TH135 and 104 genome revealed that strain TH135-specific *mce* genes, MAH_0587 and MAH_0800, are absent in the strain 104 genome due to frameshifting (Table 2). Furthermore, five genes (MAH_0796 to MAH_0799, and MAH_0801) encoding Mce family proteins in the strain TH135 genome show low homology to corresponding genes (MAV_0948 to MAV_0951, and MAV_0953) in the strain 104 genome (Table S4). Interestingly, these five *mce* genes in the strain TH135 genome were located in specific region SR-2 (Table 2).

**Table 2:** Candidates of strain-specific virulence factors in the strain TH135 genome.

Region^a^	Location (position)	Locus tag	Description	Species with most similar sequence	aa sequence identity (%)
SR-1	20756–23854	MAH _0016	MmpL family protein	*M. rhodesiae*	97
SR-2	779143–782040	MAH _0778	MmpL5 protein	*M. paratuberculosis*	96
	801455–802618	MAH _0796	virulence factor Mce family protein	*M. parascrofulaceum*	68
	802615–803649	MAH _0797	virulence factor Mce family protein	*M. kansasii*	74
	803642–804766	MAH _0798	virulence factor Mce family protein	*M. colombiense*	73
	804763–806271	MAH _0799	virulence factor Mce family protein	*M. colombiense*	70
	806268–807449	MAH _0800	virulence factor Mce family protein	*M. intracellulare*	68
	807449–809221	MAH _0801	virulence factor Mce family protein	*M. colombiense*	59
SR-3	1011402–1012598	MAH _1006	PPE family protein	*M. avium* subsp. *avium*	100
SR-10	4792912–4793808	MAH _4495	Mn^2+^ dependent Catalase	*M. parascrofulaceum*	99
	4799776–4800207	MAH _4505	MmpS4_1 protein	*M. parascrofulaceum*	99
	4800204–4803122	MAH _4506	MmpL5_5 protein	*M. parascrofulaceum*	99
Other	167053–168243	MAH _0168	PPE family protein	*M. paratuberculosis*	100
	597293–599017	MAH _0587	virulence factor Mce4F protein	*M. avium* subsp. *avium*	100
	1754780–1756012	MAH _1657	PPE family protein	*M. avium* subsp. *avium*	100
	2061187–2062653	MAH _1946	PPE family protein	*M. paratuberculosis*	99
	3644765–3647596	MAH _3375	MmpL6 protein	*M. paratuberculosis*	67
	4561515–4565480	MAH _4283	Type VII system protein EccC3	*M. avium* subsp. *avium*	99
	4914393–4918604	MAH _4605	Type VII system protein EccC2	*M. paratuberculosis*	99

a Regions SR-1 to SR-10 indicate strain TH135-specific regions, as shown in Figure 2.

There are two sets of *mce* operons that show homology to genes belonging to *mce3* operons in *M. tuberculosis* H37Rv [40] in the strain 104 genome, and genes belonging to one set showed high sequence similarity to *mce* genes (MAH_1680 to MAH_1685) in the strain TH135 genome (Table S4), while genes belonging to the other set (MAV_2532 to MAV_2537) were located in strain 104-specific region SR-19 (Table 3). The significance of the presence of these two sets of *mce* operons is an intriguing question. Furthermore, MAV_1807, a *mce* homologue, is located in region SR-16, and an operon consisting of six genes (MAV_5047 to MAV_5052), which is not present in TH135, is in region SR-21. Thus, strain 104 harbors more genes encoding strain-specific Mce proteins than strain TH135. Elucidating the roles of *mce* related genes is necessary to understand their relationships with pathological manifestations of MAC disease.

**Table 3:** Candidates of strain-specific virulence factors in the strain 104 genome.

Region^a^	Location (position)	Locus tag	Description	Species with most similar sequence	aa sequence identity (%)
SR-14	753228–754160	MAV_0790	PPE family protein	*M. colombiense*	52
	786286–787950	MAV_0828	17 kDa surface antigen family protein	*M. xenopi*	97
SR-16	1802421–1803848	MAV_1807	virulence factor Mce family protein	*M. phlei*	73
SR-19	2566171–2567511	MAV_2532	virulence factor Mce family protein	*M. intracellulare*	97
	2567550–2568578	MAV_2533	virulence factor Mce family protein	*M. intracellulare*	99
	2568575–2569900	MAV_2534	virulence factor Mce family protein	*M. intracellulare*	99
	2569924–2571243	MAV_2535	virulence factor Mce family protein	*M. intracellulare*	99
	2571240–2572400	MAV_2536	virulence factor Mce family protein	*M. intracellulare*	99
	2572402–2573877	MAV_2537	virulence factor Mce family protein	*M. intracellulare*	99
SR-21	5193791–5194750	MAV_5047	putative Mce family protein	*M. massiliense*	70
	5194747–5195673	MAV_5048	putative Mce family protein	*M. massiliense*	72
	5195745–5196863	MAV_5049	putative Mce family protein	*M. abscessus*	67
	5196863–5197834	MAV_5050	putative Mce family protein	*M. abscessus*	77
	5197822–5198829	MAV_5051	putative Mce family protein	*M. abscessus*	76
	5198826–5199770	MAV_5052	putative Mce family protein	*M. abscessus*	73
Other	889775–891037	MAV_0948	virulence factor Mce family protein	*M. paratuberculosis*	99
	891034–892056	MAV_0949	virulence factor Mce family protein	*M. avium* subsp. *avium*	99
	892056–893174	MAV_0950	virulence factor Mce family protein	*M. avium* subsp. *avium*	100
	893171–894598	MAV_0951	virulence factor Mce family protein	*M. avium* subsp. *avium*	99
	895904–897631	MAV_0953	virulence factor Mce family protein	*M. paratuberculosis*	99
	114858–115163	MAV_0117	PE family protein	*M. avium* subsp. *avium*	99
	115166–116839	MAV_0118	PPE family protein	*M. avium* subsp. *avium*	97
	1304443–1305594	MAV_1347	PPE family protein	*M. avium* subsp. *avium*	99

a Regions SR-14 to SR-21 indicate strain 104-specific regions, as shown in Figure 2.

### ESX System

Mycobacteria use type VII secretion systems (ESX-1 to ESX-5) to secrete the 6-kDa early secreted antigenic target (ESAT-6), its protein partner the 10-kDa culture filtrate protein (CFP-10), and other effector proteins such as those with conserved N-terminal domains containing proline-glutamic acid (PE) or proline-proline glutamic acid (PPE) motifs. These effectors play an important role in long-term survival of bacteria in host cells [41], [42]. Comparative genome analysis revealed that the genomes of strain TH135 and 104 contained CDSs having a 43.9% to 93.6% homology to Esx-related proteins encoded by the *esx-2* to *esx-5* loci, but not the *esx-1* loci, in the *M. tuberculosis* H37Rv genome (Table S4). There are a few differences in *esx-2* and *esx-3* loci between the strains: the strain 104 genome harbors point shift mutations in the regions corresponding to MAH_0168, MAH_4605, and MAH_4283 of strain TH135, and these regions show a respective homology of 70.1%, 88.7%, and 85.6% to PPE69, EccC2, and EccC3 in *M. tuberculosis* H37Rv (Table S4).

Mycobacteria carry many genes encoding PPE and PE proteins with unknown function. We found three PPE family protein genes (MAH_1006, MAH_1657, and MAH_1946) in the strain TH135 genome, but not in the strain 104 genome (Table 2). On the other hand, strain 104 harbors three strain-specific PE and PPE family genes (MAV_0117 encoding a PE protein, and MAV_0790 and MAV_0117 encoding PPE proteins).

### MmpL and MmpS Proteins

The high content of lipids, such as mycolic acids, in the cell wall is a distinctive characteristic of mycobacteria [43], and waxy cell walls play a pivotal role in host survival. MmpL and MmpS have been reported to mediate the transport of lipid metabolites to biosynthesize cell wall lipids [44]–[46], albeit by an undefined mechanism. The *mmpL* genes are homologous to the genes encoding proteins that belong to a family of multidrug resistance pumps termed resistance nodulation cell division (RND) [47], [48]. MmpS proteins possess one N-terminal transmembrane domain with an extracytoplasmic C-terminus [46]. The strain TH135 genome harbors all *mmpL* and *mmpS* genes found in the strain 104 genome as well as strain TH135-specific *mmpL* and *mmpS* genes, *mmpL5* (MAH_0778), *mmpL5_5* (MAH_4506), *mmpL6* (MAH_3375), *mmpL* family gene (MAH_0016), and *mmpS4_1* (MAH_4505) (Table 2). Gene *mmpS4_1* is located adjacent to *mmpL5_5* within the strain TH135-specific region SR-10, suggesting that these genes work in a collaborative manner. In addition, MAH_0016 and MAH_0778 are in region SR-1 and region SR-2, respectively. The fact that the strain TH135 genome has more *mmpL* and *mmpS* genes than the strain 104 genome suggests differences in cell wall lipid composition between the strains.

### Catalase

Mycobacteria produce catalase to remove reactive oxygen species produced by host cells to ensure their survival after invading. *M. tuberculosis* and *M. bovis* carry *katG*-gene encoding catalase, which is an important determinant of their pathogenicity in mice and guinea pigs [18], [49]. The *katG* gene also exists in the genomes of strains TH135 and 104. Interestingly, there is an additional catalase gene (MAH_4495) exclusive to the strain TH135 genome, and this gene is located in TH135-specific regions SR-10 (Table 2). Product of MAH_4495 may be involved in intracellular replication of strain TH135, although further studies are needed to determine it precise function.

### Prediction of the Bacterial Factors that Mediate Pulmonary and Disseminated MAC Disease

*M. avium* strains that cause pulmonary disease are thought to be acquired via the respiratory route and invade through the respiratory mucosal membrane. Such strains are incorporated by phagocytosis and survive in alveolar macrophages where they proceed to cause pulmonary disease [50]. In particular, the ability to replicate in professional phagocytes such as alveolar macrophages is crucial for the long-term survival of *M. avium* in lung tissue, and this appears to influence the establishment of chronic pulmonary disease. Our findings that strain TH135 specifically carries genes associated with bacterial survival in host cells, namely genes encoding MmpL and MmpS, or catalase, may be of particular importance for the establishment of pulmonary disease.

On the other hand, strains that cause disseminated disease are most likely acquired via the gastrointestinal route [50]. More precisely, bacteria are acquired through the consumption of contaminated water or food, and they experience a number of environments during the course of infection; they endure the acidic pH of the stomach, reach the intestinal lumen, and then invade lining cells of the small intestine, especially the enterocytes of the terminal ileum. Bacteria that invade the lamina propria survive after phagocytosis by phagocytic cells and spread to the blood through lymphatic vessels before being taken up by the spleen and the liver. In these processes, bacterial invasion of lining cells of the small intestine is a crucial step in the establishment of disseminated disease in immunocompromised hosts, and strain 104-specific *mce* genes associated with cell invasion and bacterial survival in cells may play an important role in the invasion of intestinal epithelial cells. McGarvey and Bermudez reported that *M. avium*, which causes disseminated disease, exhibit higher cell invasion capability than *M. intracellulare*, which does not cause disseminated disease [51]. Once functions of strain-specific genes are revealed, their roles in bacterial resistance against host defense will be elucidated in the future. This will lead to identification of bacterial factors associated with the pathological manifestations of MAC disease, which is of great significance in clinical applications.

### Screening of Clinical Isolates for Genes Located in the Strain-specific Regions

To investigate the importance of genes in strain-specific regions, we screened 35 clinical isolates (including strain TH135) from HIV-negative patients with pulmonary MAC disease and 29 clinical isolates (including strain 104) from HIV-positive patients with disseminated MAC disease for these genes (Table 4 and Table S5). As shown in Table 4, MAH_2592 in the strain TH135-specific region SR-5 was found in 28.6% of isolates from pulmonary MAC disease patients but was absent in those from HIV-positive patients. The detection rate of MAH_1001 and MAH_4506 in region SR-3 and region SR-10, respectively, was significantly higher in clinical isolates from pulmonary MAC disease patients than in those from HIV-positive patients. MAH_0016 in region SR-1 was found exclusively in strain TH135. For genes in the strain 104-specific regions, MAV_1807 in region SR-16 was found in 20.7% of isolates from HIV-positive patients but was absent in those from pulmonary MAC disease patients. Although not statistically significant, the detection rate of MAV_2532 in region SR-19 was higher in isolates from HIV-positive patients than in those from pulmonary MAC disease patients. MAV_0264 and MAV_0482 in region SR-11 and region SR-12, respectively, were found exclusively in strain 104.

**Table 4:** The presence of strain TH135- and 104-specific genes in *M. avium* isolates.

Strain	Region^a^	GC%^b^	Strain-specific gene (Locus tag)	TH^c^n = 35	HIV^d^n = 29	P value
TH135	SR-1	66.0	MAH_0016	1 (2.9%)	0 (0%)	1.000
	SR-2	63.3	MAH_0798	13 (37.1%)	5 (17.2%)	0.099
	SR-3	70.2	MAH_1001	33 (94.3%)	18 (62.1%)	0.002
	SR-5	64.1	MAH_2592	10 (28.6%)	0 (0%)	0.001
	SR-9	64.6	MAH_3208	13 (37.1%)	6 (20.7%)	0.174
	SR-10	68.2	MAH_4506	18 (51.4%)	7 (24.1%)	0.039
104	SR-11	68.9	MAV_0264	0 (0%)	1 (3.4%)	0.453
	SR-12	65.9	MAV_0482	0 (0%)	1 (3.4%)	0.453
	SR-14	66.8	MAV_0828	0 (0%)	2 (6.9%)	0.201
	SR-16	65.2	MAV_1807	0 (0%)	6 (20.7%)	0.006
	SR-19	65.0	MAV_2532	2 (5.7%)	4 (13.8%)	0.398
	SR-21	64.1	MAV_5049	8 (22.9%)	9 (31.0%)	0.573

a Lanes SR-1 to SR-10 in strain TH135 and lanes SR-11 to SR-21 in strain 104 indicate the.

strain-specific regions shown in Figure 2.

b GC% in each region.

c Strains from the sputa of patients with pulmonary MAC disease.

d Strains from the blood of HIV-positive patients with disseminated MAC disease.

Thus, screening of clinical isolates for genes located in the strain-specific regions revealed that the detection rates of strain TH135-specific genes were generally high in clinical isolates from pulmonary MAC disease patients. On the other hand, the detection rates of strain 104-specific genes were generally high in clinical isolates from HIV-positive patients. These results suggest that the genes located in the strain-specific regions have a strong influence on the pathological manifestations of MAC disease. Further study is needed to investigate the relationship between MAC disease and other specific genes, in addition to the virulence-associated genes.

In conclusion, comparative genome analysis and screening of clinical isolates for specific genes showed that the *M. avium* subsp. *hominissuis* strains which cause pulmonary and disseminated disease possess genetically distinct features. Furthermore, it is thought that the acquisition of specific genes during strain evolution has played an important role in the pathogenesis of *M. avium*. In particular, strain TH135-specific virulence-associated genes may be involved in not only the establishment of pulmonary disease, but also the pathogenicity of *M. avium*. Therefore, comparing the presence of these specific genes between *M. avium* strains isolated in Japan and those isolated abroad is important to elucidate the prevalent genetic features of *M. avium* in each country. This may also lead to the discovery of factors promoting the spread of pulmonary disease caused by *M. avium*.

## Supporting Information

Table S1 **Primers used for detection of specific genes in M. avium clinical isolates.**
(DOC)

Table S2 **List of strain TH135-specific regions and CDS found in each region.**
(XLS)

Table S3 **List of strain 104-specific regions and CDS found in each region.**
(XLS)

Table S4 **Comparison of mce- and esx-related genes in M. tuberculosis H37Rv, M. avium TH135, and M. avium 104 genomes.**
(XLS)

Table S5 **The presence of strain TH135- and 104-specific genes for M. avium isolates, including 63 clinical strains and strain 104.**
(XLS)
