# Supplementary material for: Comparative Genome Analysis of Mycobacterium avium Revealed Genetic Diversity in Strains that Cause Pulmonary and Disseminated Disease
Source: PLoS One. 2013 Aug 21;8(8):e71831. doi: 10.1371/journal.pone.0071831 (PMC3749206; doi:10.1371/journal.pone.0071831)
Supplement: Table S1 — Primers used for detection of specific genes in M. avium clinical isolates. (DOC) [file pone.0071831.s001.doc]

Table S1. Primers used for detection of specific genes in *M. avium* clinical isolates.

| Target gene | Product size (bp) | Nucleotide sequence |
| --- | --- | --- |
| MAV_0264  MAV_0482  MAV_0828  MAV_1807  MAV_2532  MAV_5049  MAH_0016  MAH_0798  MAH_1001  MAH_2592  MAH_3208  MAH_4506 | 785  808  559  995  748  755  848  1208  694  703  701  1291 | F: 5’-TTTCGTGTGCGGTGTTATCG-3’  R: 5’-GCAGAACCTGAGCCTGGAGA-3’  F: 5’-GGCGAGGTTTTCTTCGAGGT-3’  R: 5’-ACTTGGCCGTTACTCGTTGG-3’  F: 5’-TGTCGTCGAACTCCAACTCGA-3’  R: 5’-TATTCGCGTTCGGACAGGTCG-3’  F: 5’-TCTGAAGAACGGCGATGTCA-3’  R: 5’-CGGATGAAGGGTCGTACTCG-3’  F: 5’-CCGTGTTCCAGAACCTTGTC-3’  R: 5’-GTGACCGGCAGGTAGTCGA-3’  F: 5’-ACGGCGTAGGGTTCTCCATT-3’  R: 5’-TCCCTGGAACTCGTCGGTAA-3’  F: 5’-AGCGCCGCGGTAACGACTCT-3’  R: 5’-GTAGCGGCTGATCAGGAAGA-3’  F: 5’-GGTTTCATCGGCGTTGTACT-3’  R: 5’-AAAAAGCGGTGATCGTCTTC-3’  F: 5’-ACCACGTCCAAAATGCGTTC-3’  R: 5’-AACTGACACCGGCCTACGAA-3’  F: 5’-GCTACGTCTGCCTGGTCTTC-3’  R: 5’-CGGGTAAGGAAGTTGAGCTG-3’  F: 5’-TCTGACCCAACGCTGGACTT-3’  R: 5’-GGGTTCGCGAAGTCGAATC-3’  F: 5’-AGGACCGAGAAACCGCGTAT-3’  R: 5’-GCGTCATGGGTGATGATGAA-3’ |
|  |  |  |
